# A new paradigm in respiratory hygiene: increasing the cohesivity of airway secretions to improve cough interaction and reduce aerosol dispersion

**DOI:** 10.1186/1471-2466-5-11

**Published:** 2005-09-02

**Authors:** Gustavo Zayas, John Dimitry, Ana Zayas, Darryl O'Brien, Malcolm King

**Affiliations:** 1 Mucobiology Research Unit, Pulmonary Research Group, University of Alberta, Edmonton, Canada; 2 Summer students, University of Alberta, Edmonton, Canada

## Abstract

**Background:**

Infectious respiratory diseases are transmitted to non-infected subjects when an infected person expels pathogenic microorganisms to the surrounding environment when coughing or sneezing. When the airway mucus layer interacts with high-speed airflow, droplets are expelled as aerosol; their concentration and size distribution may each play an important role in disease transmission. Our goal is to reduce the aerosolizability of respiratory secretions while interfering only minimally with normal mucus clearance using agents capable of increasing crosslinking in the mucin glycoprotein network.

**Methods:**

We exposed mucus simulants (MS) to airflow in a simulated cough machine (SCM). The MS ranged from non-viscous, non-elastic substances (water) to MS of varying degrees of viscosity and elasticity. Mucociliary clearance of the MS was assessed on the frog palate, elasticity in the *Filancemeter *and the aerosol pattern in a "bulls-eye" target. The sample loaded was weighed before and after each cough maneuver. We tested two mucomodulators: sodium tetraborate (XL"B") and calcium chloride (XL "C").

**Results:**

Mucociliary transport was close to normal speed in viscoelastic samples compared to non-elastic, non-viscous or viscous-only samples. Spinnability ranged from 2.5 ± 0.6 to 50.9 ± 6.9 cm, and the amount of MS expelled from the SCM increased from 47 % to 96 % adding 1.5 μL to 150 μL of XL "B". Concurrently, particles were inversely reduced to almost disappear from the aerosolization pattern.

**Conclusion:**

The aerosolizability of MS was modified by increasing its cohesivity, thereby reducing the number of particles expelled from the SCM while interfering minimally with its clearance on the frog palate. An unexpected finding is that MS crosslinking increased "expectoration".

## Background

Infectious respiratory diseases are a prime cause of morbidity, mortality and health system utilization worldwide, but have a greater impact on the developing and least developed countries. Respiratory infections including tuberculosis (TB) caused 5.5 million deaths during 2001. Mortality rates are only part of the burden, since individuals with infections are often unable to work, becoming trapped in a vicious cycle of ill health and poverty [1].

Viruses and bacteria cause most of the infectious respiratory diseases. These pathogens can travel from country to country in different continents in a matter of hours, as exemplified by the recent outbreak of SARS [1,2]. Children, the elderly, and those with defective immune systems or with underlying chronic diseases are at highest risk of being affected by airborne infectious diseases.

Health care providers are at risk of acquiring transmissible respiratory diseases while providing care or working in contaminated environments. The SARS outbreak has validated these concerns in advance of the feared flu pandemic. SARS has also confirmed that infectious diseases can cause great disruptions in all sectors – economic, educational, recreational, familial – and can bring health care systems to near collapse. Several strategies and devices have been designed to protect various sectors of society whether at peacetime or during conflicts.

Different individual protective barriers, such as surgical masks, face shields and respiratory protection equipment have been used; however, they yield a much lower protection than the theory might indicate [3]. The individual protective equipment must be chosen according to the agent and to the activities performed by the user, and consider the compliance of the user and the adequacy of the fit and the effectiveness of the seal. Wearing well-fitted protective equipment for long periods of time could impair the satisfactory performance of duties of health care providers. Hence adequate and cost-effective strategies are still needed.

Vaccination is the main strategy to control outbreaks of a number of infectious diseases including the flu pandemic, but the WHO states that other advance control measures will definitively be required since vaccines take time to become available [4].

Tuberculosis (TB) caused by *Mycobacterium tuberculosis *is a curable, transmissible malady known for at least 3,400 years. The WHO and other organizations estimate that two *billion *people around the world are infected with *M. tuberculosis*. Eight million new active cases of TB are reported annually, mostly in age-productive adults. Two million people died of TB in 2002, more than in any previous year in history, becoming the leading killer of adults worldwide [5].

*M. tuberculosis *can develop resistance to multiple, first-line anti-TB drugs (MDR-TB). This type of resistance spirals the costs beyond 100 times per patient, bringing more suffering to countries afflicted with TB [6]. Involuntary quarantine in TB patients with non-adherent attitudes is enforced as a last resource measure [7] to control its spread.

In cystic fibrosis, recurrent and persistent respiratory infection by *Pseudomonas aeruginosa *is a major cause of morbidity and mortality in CF patients. Jones *et al. *[8] concluded that cross-infection outbreaks of *P. aeruginosa *were caused by respiratory aerosol dissemination.

Infectious respiratory diseases, whether viral or bacterial, are transmitted to non-infected subjects when an infected patient expels pathogenic microorganisms to the surrounding environment when coughing, sneezing or even possibly when talking. Duguid [9] determined that the number of particles of 100 μm or less in diameter contained in the aerosol formed during speaking were between 0 – 200 particles, while during coughing there were 0 – 3,500 particles and during sneezing there were 4,500 – 1, 000,000 particles. Fennelly [10], studying the size of the infectious particles generated during cough, estimated that close to 50% of them were fine particles, less than 2 μm range, small enough to reach the alveoli within the lungs.

For the transmission of any respiratory pathogen at least three components are required: a) the transmissor or source (infected person), b) the surrounding environment, and c) the recipient (non-infected person). It is important to additionally consider the concentration of infectious particles determined by the volume of the space and its ventilation, the length of time of exposure, as well as the status of defense mechanisms of the exposed individual.

This prompted a research question: Is it possible to control the transmission of infectious respiratory diseases by modulating the physical characteristics of viscoelasticity, cohesivity and surface tension of the respiratory secretions to minimize the aerosolization that facilitates transmission of airborne diseases – a transmission-blocking approach? We found in our literature search no report in regards to this type of approach or drugs. Before testing the mucomodulators we established a fundamental criterion: Can we modulate the physical characteristics of the respiratory secretions to reduce aerosolization without affecting normal clearance function, or even better by improving it?

**Figure 1:**
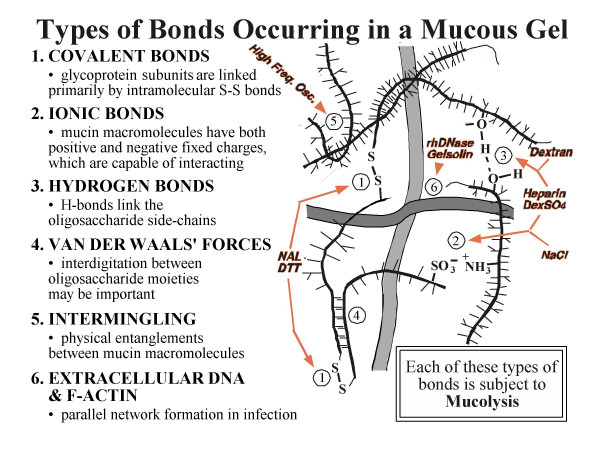
Schematic diagram of the macromolecules of mucus with the different types of crosslinks. Sodium tetraborate would contribute to crosslink formation involving accessible galactose moieties in the oligosaccharide side-chains. Calcium chloride would add crosslinking through divalent ionic interactions.

Hence, we focused our attention on the mucus layer of the airways. The mucin macromolecule (Figure 1) consists of a protein core surrounded by short oligosaccharide side-chains, held together by different links: O-glycosidic bonds, disulphide bridges, hydrogen bonds and ionic bonds, which are the targets of the existing mucolytic agents [11].

The mucus along with serous fluid forms the airway surface fluid (ASF) that provides a protective milieu for the airways. The composition and physical characteristics of ASF allow for normal ciliary activity and airway protection [12,13]. When disruption of normal secretory or mucociliary clearance processes occurs, respiratory secretions can accumulate and impair pulmonary function, reduce lung defenses and increase the risk for infection and possibly neoplasia [14].

Ninety-five per cent (95%) of mucus is water and the remaining 5% is made up of proteins, carbohydrates, salts, etc. Therefore, when the airway mucus layer interacts with high-speed airflow during coughing, there is formation of droplets of different sizes that are expelled to the surrounding environment as an aerosol. The concentration of droplets and their size distribution may each play an important role in the transmission of respiratory infectious diseases.

Micron-sized droplets dry quickly, remain airborne for long periods and reach the lower respiratory system when inhaled. These breathable particles might indirectly infect many persons, e.g. via a common ventilation system. In contrast, large droplets are propelled onto the nearest surface – oral, respiratory, or ocular mucosa of a nearby person – or settle readily to the floor. Regarding transmission, larger particles may have an immediate effect – direct but limited – while micron-size particles may have more indirect effects with longer-lasting and widespread consequences.

For this study, our basic goal was to assess a new generation of mucomodulators in a novel approach that has the potential to modulate the physical characteristics of the mucin glycoprotein gel network. This intervention would increases the mucin crosslinking binding sites to reduce the aerosolizability on mucus simulants (surrogate of transmissibility), and/or forming poorly soluble mucin complexes. We aimed to achieve the former while interfering only minimally with normal mucus clearance and hopefully even improving clearability in patients with airway mucus retention.

Our hypothesis is that transmission of airborne diseases can be controlled by implementing a co-adjuvant pharmacological intervention, additional to the indicated standard treatment, aimed at modulating the physical and biochemical characteristics of the respiratory secretions of both the transmissor who carries the infection to minimize expectorated material that carries the pathogens, and in the non-infected potential recipient.

## Methods

For this phase we used mucus simulants for testing the effect of the mucomodulators. We also used our *ex vivo *frog palate exposure model in combination with our simulated cough machine to assess the clearability as well as the aerosol formation capability of the artificial mucus with or without mucomodulators.

### Mucus simulants (MS)

The MS ranged from a non-viscous, non-elastic substance (water) to MS of varying degrees of viscosity and elasticity. To prepare viscoelastic mucus simulants, we followed the procedure described by King *et al*. [15]. Mucus simulants were formulated and developed to be transported within the normal range of velocity in a ciliated epithelium and cleared from a simulated cough machine (SCM) set to have a close to normal human cough. Mucus simulants viscous-only were prepared dissolving different concentrations (0.5 %, 0.75 % and 1 %) of locust bean gum (LBG) in warm Ringer solution, and then adding activated charcoal, bromophenol blue 1% or Coomassie blue to dye the solution for better visualization.

### Frog palate preparation

From a bullfrog, *Rana catesbiana*, the upper portion of the head was removed following the procedures described in previous work [16,17], by cutting with scissors through from the junction of the posterior pharynx and esophagus out to the skin of the back. This procedure was carried out after lowering the body temperature of the frog for 30 – 60 minutes inside a refrigerator to abolish pain sensations. The palate was examined for macroscopic lesions, such as ulcers, petechia or redness as evidence of inflammation; only palates free of inflammatory indicators were used. Any blood remaining in the epithelial surface was carefully washed away, then the excised head was placed palate side facing upwards on a piece of gauze saturated with frog Ringer solution (FRS) in a Petri dish.

The palate was placed inside the frog chamber, a wooden box with a glass top and fitted glass front, and manipulated through glove openings. The humidity inside the box was maintained near 100% using a Pari jet nebulizer, and the temperature was kept between 22° and 24°C by a rheostat-controlled, externally mounted light source. Before carrying out any measurement, the palate was allowed to stabilize inside the box for 15 minutes before testing.

The FRS was prepared by mixing standard Ringer injection with sterile water (2:1). The composition of standard frog Ringer (in mmol/L) is 90 NaCl, 3 KCl, 2 CaCl2, and 15 NaHCO3 (220 mosm/L). The Health Sciences Animal Policy and Welfare Committee, University of Alberta approved the experimental procedures involving animals

### Simulated mucus transport velocity (MTV) determination

The palate was placed under a dissecting stereomicroscope provided with a reticulated eyepiece. Mucociliary clearance was determined by observing the movement of particles of charcoal powder gently deposited on a sample of simulated mucus on the palate surface; its clearance was visually monitored and MTV determined. The displacement of 3 – 5 μL of endogenous frog mucus sample was calculated by dividing the distance traveled by the transit time across the 0.3-inch (7.62 mm) segment marked between 0.1 and 0.4 inches in the graduated eyepiece. To obtain control and simulated mucus transport velocities, at least five measurements of the time required for the mucus sample to travel the defined distance were made.

### Tissue and mucus sample collection

Samples of tissue and mucus from frog palates exposed to topical application of mucomodulators, were immersed in glutaraldehyde 2.5%, and stored at 4°C for later scanning electron microscope (SEM) studies. The epithelial tissue was carefully dissected and separated from the palate musculature.

### Aerosol formation assay

To assess the aerosol pattern formed after coughing, we used a range of fluids of varied physical characteristics from non-viscous, non-elastic substances such as water, to highly viscoelastic substances such as simulated mucus samples of varying degrees of viscosity and elasticity. To better visualize the dispersion pattern of the fluids exposed to high-speed airflow in the SCM, we stained all the fluids.

### Spinnability

The capacity of a fluid to form threads when stretched, an expression of elasticity, was assessed in the Filancemeter (SEFAM, France) prior to exposure to airflow in the SCM. A volume of approximately 50 μL of the sample to be studied is introduced in a reservoir that is then sucked in. A low current is applied to the sample from a voltage supply, and when the sample is stretched vertically, the apparatus electronically measures the maximum length of the thread at the moment of the rupture of the thread. For every mucus sample, four measurements of the length of the thread at the moment of rupture were made, and used to compute the spinnability in mm.

### Cough aerosolization

The simulated cough machine was used to test the main hypothesis that the modulated mucus will result in less aerosolized (airborne) material following cough. Since cough is the most critical process of mucus aerosolization, the successful reduction of droplet production during cough will likely predict reduced amounts emitted during sneezing and speaking. All fluids were placed at different distances from the "mouth" opening of the simulated airway to examine if distance from the target may be a factor in determining the dispersion pattern. Later, we decided on a fixed distance to place the sample of MS to be exposed to high-speed airflow interaction. The target was a "bullseye-type" (circles within circles). We also placed paper sheets to lateral sides, on top, and below the target sheet to account for any fluid that is dispersed in areas other than on the target sheet.

### Cough clearability assay

The modified simulated cough machine system comprises the following elements: A 10-L tank with compressed air, which serves as a pressure reservoir that generates airflow, simulating the lungs during a cough maneuver. The pressure generated prior to a normal adult cough is approximately 8 psi, and we used this pressure value to simulate the airflow pattern of a human cough for each trial.

This modified machine has a model "*airway or trachea*" consisting of a 28 cm long rigid acrylic tube with a circular 2.5 cm cross-section. It has an 8 cm detachable muzzle end-piece (59.805 g) that enabled us to weigh the sample of MS loaded before and immediately after each cough maneuver and report the mass of MS expelled or retained. A solenoid valve located between the pressure reservoir and the model trachea control the gas released from the tank.

An aliquot of sputum is layered on the bottom of the model trachea and driven forward by the pressurized gas. The distance traveled by sputum samples under standardized cough-simulating airflow is used as a measure of cough clearability. The target was placed at a working distance of 40 cm from the mouth of the SCM. The simulated cough maneuver is repeated four times with each sputum sample.

Airway *clearance *("expectoration" of mucus simulant from the "airways"): Measurements of the weight of the MS sample loaded before and after each cough maneuver were carried out in a Mettler AE 163 microbalance.

### Scanning electron microscopy

Samples of mucus and tissue were placed in 2.5% glutaraldehyde solution immediately after collection and stored at 4°C until processing. The samples were post-fixed in 1% osmium tetroxide in Millonig's buffer at room temperature for one hour. They were then washed in a series of ethanol (50 – 100%), ten minutes at each step, followed by two additional periods of absolute ethanol (10 minutes each). The samples were further dehydrated by critical point drying at 31°C for 5 – 10 minutes, then mounted on a specimen holder for SEM and dried overnight in vacuum desiccators. In the final stage of preparation for viewing, the samples were sputter coated with gold (Edwards, model S150B Sputter Coater). Samples were viewed using SEM (Hitachi S-2500). Images were scanned directly to a computer and stored as image files for subsequent viewing and analysis.

### Mucomodulators

We explored four different approaches that have the potential to accomplish our primary goals of reducing aerosolization of mucus while promoting airway clearance. However, for the purpose of this study, we report only two of them here. Sodium tetraborate, named also as XL"B" or mucomodulator B, and calcium chloride, named also as XL "C" or mucomodulator C. The other approaches, high molecular weight dextran, named also XL "D" or mucomodulator D, and polyarginine, named also XL "A" or mucomodulator A will be reported independently in other publications [18].

### Sodium tetraborate (XL "B")

Sodium tetraborate causes reversible crosslink formation between galactose units [19], which are the major neutral sugar components of mucin. Addition of borate to galactose polymers preferentially raises elasticity relative to viscosity. We added three different volumes of 0.1 M borate (1.5 μL, 15 μL and 150 μL) to the MS to produce three levels of crosslinking, and exposed each of the gels to four cough maneuver measurements per trial.

### Calcium chloride (XL "C")

Calcium chloride increases the concentration of divalent cations in the mucus. Conceptually, this is the opposite of what occurs in nature during mucin exocytosis, where intracellular mucin granules, which were held tightly through ion crosslinks, give way to much looser interactions as ion exchange occurs during fusion with the apical membrane [20]. A volume of 4 μL of frog Ringer and 4 μL of three different concentrations of calcium chloride (0.001 M, 0.01 M and 0.1 M) were directly applied through a micropipette to a fresh frog palate in order to assess their effect on mucus transport velocity and on the morphology of the tissue and mucus layer.

### Statistical analysis

Data are expressed as mean ± standard deviation unless otherwise stated. A paired Student-T test was used for simple comparison. Neuman-Keuls analysis was applied for repeated measurements. The level of significance was set at 5 %.

## Results

In this study we report the effect that the mucomodulator XL"B" has on MS on spinnability, clearance by ciliary action and by high-velocity airflow interaction (cough maneuver), as well as some effects that XL "C" has on the physical appearance of frog mucus and on native mucus transport on the frog palate.

### Effects of sodium tetraborate

#### Staining the samples

Bromophenol Blue made the MS solutions very watery and it reduced viscosity of the MS; hence we rejected the use of this dye from further testing. Activated charcoal did not alter the viscoelastic properties of the MS sample and helped to better visualize the gel sample on the frog palate; however charcoal adds mass to the sample that eventually might alter the dispersion pattern. Coomassie Blue did not alter the viscoelastic properties of MS; hence it was used to dye the samples.

#### Transportability

Samples of MS of non-viscous, non-elastic properties (frog Ringer solution) of approximately 0.2 – 0.5 mL at room temperature were placed on the frog palate; these dispersed on the surface and were not transported by ciliary action.

Viscous-only samples in the low range of concentration (LBG 0.5%) did not maintain their shape and dispersed on the epithelial surface and were not propelled by ciliary action. Viscous-only samples in a higher concentration range (LBG 1.0%) maintained their shape and were transported very slowly by ciliary action, approximately five times slower than control.

#### Thread formation

**Figure 2:**
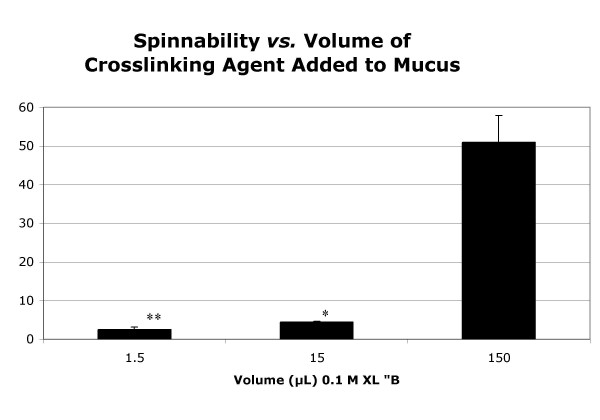
Effect of the crosslinking agent XL "B" (sodium tetraborate) on spinnability in artificial mucus. * p < 0.05 with respect to 150 μL; ** p < 0.01 with respect to 150 μL.

Non-viscous, non-elastic samples and the viscous-only samples did not produce any measurable spinnability in the Filancemeter. Working with samples of viscoelastic MS, we attained repeatable lengths of the filament in the low range (1 – 10 mm) and high range (30 – 50 mm) values in the Filancemeter, but found it difficult to consistently obtain medium ranges (11 – 29 mm), even using different combinations of viscosity and elasticity in MS. Elasticity of the crosslinked MS (length of the filament) increased in a direct proportion to the volume (1.5 μL, 15 μL and 150 μL) of 0.1 M added borate (Figure 2).

Paired T-testing showed significant differences in thread formation when comparing the three added volumes. The Newman-Keuls test also indicated a significant difference (p < 0.05) in thread formation of the MS when comparing medium added volume (15 μL) to high volume (150 μL) of sodium tetraborate. Also, the Newman-Keuls test indicated a very significant difference (p < 0.01) in thread formation when comparing low added volume (1.5 μL) to high volume (150 μL) of borate.

The average linear velocity transport rate by ciliary action of crosslinked mucus simulant in the frog palate was approximately 60 percent (46% – 76%) as effective as native mucus transport used as control (100%) when different volumes (1.5 μL – 150 μL) of 0.1 M sodium tetraborate were applied. When different volumes of lower concentration borate (0.01 M) were applied, the transport velocity rate was no more than 30 percent (13% – 30%) as effective as control.

#### "Cough clearance"

Non-viscous, non-elastic MS samples (water) placed at various distances from the mouth of the artificial airway and exposed to a simulated cough maneuver had a high degree of dispersion onto the bullseye target.

**Figure 3:**
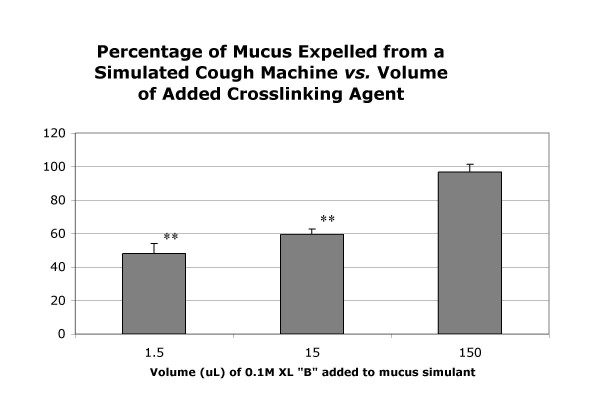
Cough clearance ("expectoration") of sodium tetraborate (XL :B") crosslinked mucous gel simulants, i.e. the percentage of initial load clearing the mouth of the artificial trachea during the cough maneuver. ** p < 0.01 with respect to 150 μL.

A sample of approximately 0.5 mL of crosslinked MS stained with Coomassie Blue was loaded inside the detachable portion of the artificial airway and then weighed in a microbalance. The cough machine was triggered and the detachable portion with the remaining aliquot of the sample not expelled by the cough maneuver was immediately weighed again. The amount of MS expelled from the artificial airway increased in direct proportion to the volume of 0.1 M sodium tetraborate added (Figure 3). Paired T-test analysis showed there exists a significant difference comparing the volume of MS sample expelled with increasing volume of 0.1 M added borate. The Newman-Keuls test indicated a significant difference (p < 0.01) in the weight of MS expelled from the SCM when comparing low added volume (1.5 μL of XL "B") to high volume (150 μL of XL "B").

**Figure 4:**
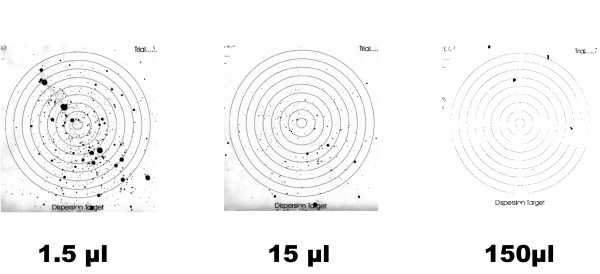
Aerosolization and dispersion pattern effects for sodium tetraborate crosslinked mucous gel simulants. Aerosol pattern at 40 cm. following a standardized cough maneuver. Both the amount of aerosol striking the target and the amount of fine aerosol decreased progressively with added crosslinking agent.

MS loaded in the simulated airway and exposed to high-speed airflow interaction (simulated cough) broke up into multiple droplets of various sizes that were expelled in the form of a cloud of aerosol that landed on the target. Some portions of the MS expelled did not reach the target but landed on pieces of paper placed below, above and along the lateral sides of the corridor between the mouth and the target. The amount of aerosol striking the target and the amount of fine aerosol decreased progressively with added volume of crosslinking agent (Figure 4).

The volume of XL "B" added to the MS sample was inversely related to the number of droplets formed after a cough maneuver and airflow interaction; the larger the volume added, the fewer droplets were formed. It was also observed that as the added volume of XL "B" (borate) increased, the size of the droplets formed after the airflow interaction increased; hence few, big cohesive droplets were formed, which did not reach the target but did fall in the paper placed immediately below the exit. The proportion of MS sample remaining in the "airway" after a cough maneuver correspondingly decreased with increasing added volume of crosslinking agent (from 53% for 1.5 μL to 40% for 15 μL to just 4% for 150 μL added sodium tetraborate).

### Calcium chloride effects

**Figure 5:**
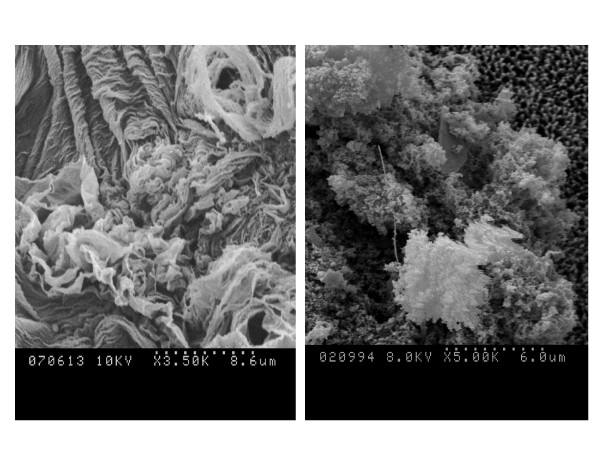
Effect of the crosslinking agent calcium chloride (XL "C") on the physical changes of the fresh frog mucus. SE micrograph showing the "clumping" effect of calcium chloride applied topically to the frog palate. The ciliated surface of the palate is seen below the mucus and does not appear different from control views.

XL "C" (calcium chloride 5 μL) applied topically to our *ex vivo *frog palate model, causes mucus "clumping" without modifying the normal physical appearance of the ciliated surface when compared with ciliated surface exposed to frog Ringer solution (Figure 5).

**Figure 6:**
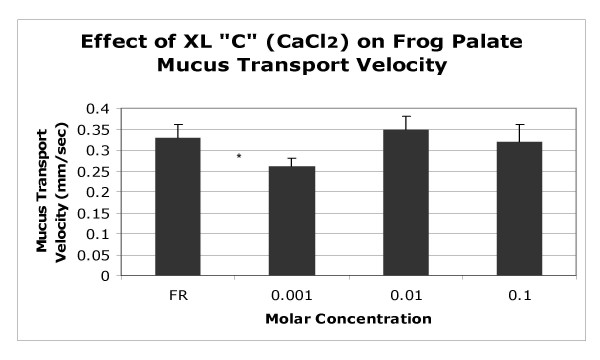
Effect of different concentrations of sodium tetraborate (XL "B'') on MTV of frog mucus. * p < 0.05 with respect to the other concentrations.

Direct application (5 μL) of three concentrations (0.001 M, 0.01 M, 0.1 M) of CaCl2 on the frog palate seems to moderately slow the way that native frog mucus is transported by ciliary action in the epithelial model (Figure 6). The Newman-Keuls analog procedure showed a significant difference (p < 0.05) of MTV of frog mucus samples only when the lowest concentration was applied, compared to the other concentrations and to the Frog Ringer control.

## Discussion

In this study we have acquired preliminary evidence that the use of mucomodulators yields acceptable mucociliary clearance in an *ex vivo *epithelial model, increases "expectoration" in a model system, and yet achieves a desired target of a significant reduction in fine aerosol formation. We have also obtained graphic evidence that one of our mucomodulator agents, calcium chloride, which increases the concentration of divalent cations, in fact **increases **the interaction of the ion crosslinks in the frog mucus, resulting in modification of the physical structure of the mucous layer. This action suggests that the aerosolizability of the mucus or the ability to form tiny droplets may be decreased.

To enhance the validity of the obtained results, we formulated an artificial mucus according to King [19] that displayed physical properties of viscosity, elasticity, reproducible thread formation and transportability in a ciliated epithelium within an acceptable range that most closely simulates airway mucus. The aerosolizability of MS was modified by increasing its cohesivity, thereby reducing the number of particles expelled from the SCM while interfering minimally with its clearance on the frog palate. An unexpected finding is that MS crosslinking increased "expectoration". Rubin & Van der Schans [21] stated, *"If an intervention is thought to have a directly measurable effect on sputum properties or on sputum clearability and if these changes cannot be demonstrated in vitro, it is unlikely that they will be observed in clinical trials."*

Results presented in this article and obtained thus far from our model system – mucous gel simulant, artificial airway, simulated cough function and *ex vivo *epithelial model – are limited but striking, and clearly support our mucomodulator approach. We were encouraged to continue with an *in vivo *mammalian model that will be presented independently (18). We expect that similar results will eventually be observed in clinical trials. Results from this study suggest that the mucomodulators tested appear not to affect the morphology or function of the mucociliary apparatus.

We have designed a new *mucomodulator therapy *– aiming to change the physical properties of ASF to enhance the clearance of mucus from the respiratory tract, as well as to optimize aspects of lung defense that depend on the mucous layer. Mucomodulators work differently than mucolytics, since the latter were designed to disrupt the structural macromolecules that give respiratory tract mucus its physical characteristics, and from other agents designed to increase mucus flow by stimulating ciliary activity or improving periciliary fluid hydration. As used here, mucomodulators act to increase the cohesivity of airway mucus simulants. Mucomodulator therapy to combat mucus retention may become a major consideration in the treatment of cystic fibrosis and other chronic lung diseases in which mucus hypersecretion and impaired airway clearance produce symptoms [11], and require adequate and novel secretion management. With the mucomodulator approach, we intend to adjust or regulate the mucin macromolecules that give respiratory mucus its physical characteristics in order to minimize the aerosolization that facilitates transmission of airborne diseases. It should be noted that it is not "normal" to cough and aerosolize particles that can be breathed in by others, although it is common. Thus a potential therapy that could reduce this tendency to aerosolize particles when coughing should be considered a form of modulation in the sense of restoring a balance or state of homeostasis.

Cellular debris and other byproducts of infection contribute to make more difficult the clearance of infected mucus in CF patients, requiring antibiotics and mucolytic therapy. These approaches have helped to reduce the mortality rate in CF and other patients; however, there still remains a long way to go to reduce transmission of pathogens in non-infected subjects.

We have invented a novel and non-intuitive approach to improving airway mucus clearance by *adding *crosslinks in a selective and controlled manner through a specialized new generation of mucomodulators. If further testing bears out our initial findings, it could lead to treatments that would reduce the rate of morbidity and improve the quality of life of individuals who have difficulties with airways secretions management.

Essentially, we have found preliminary evidence that certain mucomodulators that increase cohesive interactions between mucin macromolecules lead to a mucus that is more easily clearable, most likely by airflow-dependent mechanisms, as suggested by results obtained from our SCM The latter is a preliminary answer to a recurring question from lung experts: that using this approach could "gum up" the airway secretions. Such an improvement in mucus quality related to clearance would benefit many, if not most, patients with chronic airway disease, including those suffering from chronic obstructive pulmonary disease, who depend in whole or in part on airflow clearance mechanisms to maintain airway hygiene [13,22]. Further work is needed in clarifying the mechanisms involved in the clearance of the airway secretions.

This novel approach might be of particular importance for those in the ICU or those with spinal cord lesions who require assisted ventilation. At the same time, increasing the cohesiveness in this manner leads to a reduction in fine aerosol formation during expectoration – a clear advantage in helping to control the spread of airborne infections. This new approach may be applicable to a range of infectious disease threats occurring naturally like the feared next flu pandemic or the possible deliberate use of biological agents.

There are some additional critical advantages of this novel technology: Health Canada and the US FDA and other regulatory agencies have already approved the selected compounds for pharmaceutical use in humans. If any of these compounds (or a combination thereof) is judged suitable for clinical trials, established safety data could be utilized to fulfill some of the regulatory requirements for pre-clinical studies.

Some areas of applicability would include transmission prevention of clinically and epidemiologically important pathogens like *M. tuberculosis *and MDR-TB, in the feared flu pandemic and SARS-CoV, as well as *P. aeruginosa *in cystic fibrosis. Our mucomodulators would most likely represent containment for transmission of respiratory pathogens especially during outbreaks, allowing strategic sectors of society to be protected and remain functional, like the health system, law enforcement, service providers, as well as the work force.

Some other critical advantages of this novel technology are: Our low cost mucomodulators will not need to undergo constant development and reformulation due to viral or bacteria mutation or resistance. The technology is designed to act on the host rather than the causative organism, which will provide more longevity to it. The compounds under consideration are less perishable than vaccines, amenable to distribution, particularly in warm climates, and simple to manufacture to even allow for rapid response in outbreak situations. In addition, some of the concept testing could involve veterinary trials in airborne animal diseases, an aspect which we are also exploring.

In model studies using vegetable polysaccharides, sodium tetraborate preferentially raises elasticity relative to viscosity, and current knowledge prior to the development of mucomodulators indicated that tetraborate solutions would favour mucociliary clearability at the expense of cough clearability and aerosolizability [12] Now, it appears that cough clearability and aerosolizability are both linked to cohesivity, but in opposite senses, at least within the range of the current study. Thus it appears that sodium tetraborate solutions, by altering mucous gel cohesivity, can decrease aerosolizability (fine aerosol formation during coughing) while increasing cough clearability (bulk clearance of mucus).

In summary, the mucomodulator technology is innovative in that it represents a reversal in current thinking about respiratory disease management. The resulting product will be an unprecedented type of tool in airway hygiene, in secretion management as well as in prevention of droplet-spread illnesses, close person-to-person-contact and airborne transmission – a safe, efficacious, non-vaccine preventative therapy.

## Competing interests

The author(s) declare that they have no competing interests.

## Pre-publication history

The pre-publication history for this paper can be accessed here:


